# A comparative study of posterior cingulate metabolism in patients with mild cognitive impairment due to Parkinson's disease or Alzheimer's disease

**DOI:** 10.1038/s41598-023-41569-5

**Published:** 2023-08-30

**Authors:** Mingming Huang, Hui Yu, Xi Cai, Yong Zhang, Wei Pu, Bo Gao

**Affiliations:** 1 Department of Radiology, Affiliated Hospital of Guizhou Medical University, Guiyang, 550004 China; 2 General Practice Center and Department of Radiology, The Seventh Affiliated Hospital, Southern Medical University, Foshan, 528200 China

**Keywords:** Metabolic disorders, Neurological disorders, Neuroscience, Cognitive neuroscience, Diseases of the nervous system, Biomarkers, Prognostic markers, Parkinson's disease, Parkinson's disease, Neurology, Alzheimer's disease

## Abstract

Few comparative studies have assessed metabolic brain changes in cognitive impairment among neurodegenerative disorders, and the posterior cingulate cortex (PCC) is a metabolically active brain region with high involvement in multiple cognitive processes. Therefore, in this study, metabolic abnormalities of the PCC were compared in patients with mild cognitive impairment (MCI) due to Parkinson’s disease (PD) or Alzheimer’s disease (AD), as examined by proton magnetic resonance spectroscopy (^1^H-MRS). Thirty-eight patients with idiopathic PD, including 20 with mild cognitive impairment (PDMCI) and 18 with normal cognitive function (PDN), 18 patients with probable mild cognitive impairment (ADMCI), and 25 healthy elderly controls (HCs) were recruited and underwent PCC ^1^H-MRS scans. Compared with HCs, patients with PDMCI exhibited significantly reduced concentrations of *N*-acetyl aspartate (NAA), total NAA (tNAA), choline (Cho), glutathione (GSH), glutamate + glutamine (Glx) and total creatine (tCr), while ADMCI cases exhibited significantly elevated levels of myo-inositol (Ins) and Ins/tCr ratio, as well as reduced NAA/Ins ratio. No significant metabolic changes were detected in PDN subjects. Compared with ADMCI, reduced NAA, Ins and tCr concentrations were detected in PDMCI. Besides, ROC curve analysis revealed that tCr concentration could differentiate PDMCI from PDN with an AUC of 0.71, and NAA/Ins ratio could differentiate patients with MCI from controls with normal cognitive function with an AUC of 0.74. Patients with PDMCI and ADMCI exhibited distinct PCC metabolic ^1^H-MRS profiles. The findings suggested cognitively normal PD patients with low NAA and tCr in the PCC might be at risk of preclinical PDMCI, and Ins and/or NAA/MI ratio in the PCC should be reconsidered a possible biomarker of preclinical MCI in clinical practice. So, comparing PCC’s ^1^H-MRS profiles of cognitive impairment among neurodegenerative illnesses may provide useful information for better defining the disease process and elucidate possible treatment mechanisms.

## Introduction

Mounting evidence suggests cognitive impairment may be induced by neurodegenerative disorders^1–4^. Alzheimer’s disease (AD) is one of the most frequently diagnosed diseases impairing cognitive function in older adults^5^. Besides AD, Parkinson’s disease (PD) is also a commonly diagnosed neurological disorder^6^, and patients with PD often experience cognitive impairment, and eventually dementia^4^. However, whether cognitive impairment is caused by PD or AD, there is considerable variation between the onset of cognition decline and the occurrence of dementia^7^, and this delay provides a window for potential therapeutic interventions^2,8^. During the progressive neurodegeneration, suitable biomarkers may facilitate timely interventions to slow cognitive impairment^9^.

Proton magnetic resonance spectroscopy (^1^H-MRS)^10,11^ is a potential option for assessing neurobiomarkers of cognitive status, which may be used to evaluate brain metabolites such as *N*-acetyl-aspartate (NAA), total creatine (tCr), choline (Cho), myo-inositol (Ins), glutamine (Gln), and glutamate (Glu), and can also reflect neuronal integrity (NAA), membrane turnover (Cho), glial metabolism (Ins), energy metabolism (Cr) and glutamatergic neuronal activity (Glu or Gln) in patients. Abnormal metabolic ratios (NAA/Cr, Cho/Cr and Ins/Cr) or absolute metabolite concentrations in the PCC have been detected in AD, as well as mild cognitive impairment^12,13^. A 7-year follow-up study suggested cognitively normal elderly individuals with low NAA/Ins ratio in the PCC might be at risk of preclinical AD^14^.

Besides the aforementioned features, the PCC represents a highly connected, metabolically active brain region, which is also highly involved in multiple cognitive processes^15–17^. Multiple reports have suggested the PCC is one of the first regions to be compromised in early AD^18^. It is also affected in PD cases^19^, showing gray matter atrophy (via structural MRI)^20^, reduced perfusion (via arterial spin labeling MRI)^21^ and compromised metabolism (via positron emission tomography)^22^. A study of cerebral glucose metabolism also suggested PCC dysfunction as the primary neuroimaging feature in PD patients at risk of dementia^21^. However, the potential role of ^1^H-MRS as an in vivo molecular imaging method to confirm early and differential PDMCI diagnosis is controversial. Klietz et al.^23^ and Guan et al.^24,25^ reported altered neurometabolic profile in early onset of PD. Nie et al. suggested metabolic changes in the PCC occur at the early cognitive impairment stage in PD patients, which may be used as a marker of PDMCI^26^. On the contrary, Almuqbel et al. suggested that the metabolic ratio in PCC cannot track cognitive decline in PD in the clinical setting^9^. Weiduschat et al. also revealed that either ^1^H or ^31^P-MRS could detect metabolic abnormalities in early PD^27^.

Unlike AD, whether ^1^H-MRS of the PCC can be used as an in vivo molecular imaging technique for the early diagnosis of PDMCI and for monitoring the efficacy of therapeutic interventions is unknown. Therefore, the current study aimed (1) to detect metabolic alterations in the PCC in PD cases with or without mild cognitive impairment (PDMCI/PDN) and (2) to investigate metabolic features in MCI induced by AD, and to compare the metabolic patterns of the PCC in MCI patients between these two neurodegenerative diseases.

## Materials and methods

### Study participants

Thirty-eight patients with idiopathic PD (63.5 ± 12.1 years old including 21 males), comprising those with normal cognitive status (PDN) and mild cognitive impairment (PDMCI); 18 patients diagnosed with ADMCI (64.3 ± 8.3 years old, with 13 males) and 25 age- and gender-matched healthy controls (HCs, 56.6 ± 9.6 years old with 9 males) (Table 1) were recruited from the Affiliated Hospital of Guiyang Medical University. PD diagnosis was based on the United Kingdom Parkinson’s Disease Society Brain Bank criteria, and the patients were followed up to confirm the diagnosis. No subjects reported other neurological or psychiatric diseases, and on FLAIR scans, neither white matter degeneration nor lacunae infarction was found in any subject. All patients provided signed informed consent, and the study was approved by the Ethics Committee of the Affiliated Hospital of GuiZhou Medical University, and followed the Declaration of Helsinki.

**Table 1:** Demographic data for healthy controls, and PDN, PDMCI and ADMCI patients.

	HC	PDN	PDMCI	ADMCI	*F* value	*p* value
No (male/female)	25 (9/16)	18 (11/7)	20 (10/10)	18 (5/13)	NA	< 0.01
Age, year (mean ± SD)	56.6 ± 9.6	62.6 ± 12.1	64.6 ± 12.3	64.3 ± 8.3	2.453	0.070
Education, year (mean ± SD)	10.7 ± 2.3	10.7 ± 2.6	10.0 ± 3.8	11.3 ± 2.7	0.574	0.634
H&Y stage	NA	1.9 ± 0.8	1.9 ± 0.7	NA	NA	0.967
MMSE	29.6 ± 0.6	29.9 ± 0.3	25.4 ± 2.8	26.6 ± 3.1	23.33	0.000

*F* values calculated by simple ANOVA across groups. *p* < 0.05 was considered significant.

*HC* healthy controls, *PDN* Parkinson’s disease patient with normal cognition, *PDMCI* Parkinson’s disease patient with mild cognitive impairment, *ADMCI* patients with mild cognitive impairment, *MMSE* Mini Mental State Examination, *H&Y* Hoehn and Yahr scale, *SD* standard deviation, *NA* not applicable.

### Clinical assessments

The Mini Mental State Examination (MMSE) was used to measure global cognitive function in all subjects (Table 1). Subtype assignment in PD was based on previously established methods^4^; criteria for PDMCI followed Caviness et al. Individuals had subjective cognitive complaints, with no significant functional decline. PDMCI cases demonstrated a deficit of at least 1.5 standard deviation (SD) below the expected age-corrected mean score in one of five designated cognitive domains. These domains of potential cognitive dysfunction were frontal/executive, amnestic, visuospatial, attention, and language. According to these criteria, 18 patients were classified as PDN and 20 as PDMCI.

The 18 patients diagnosed with ADMCI were all recruited from the memory clinic of the Affiliated Hospital of GuiZhou Medical University, and structural magnetic resonance imaging (MRI) and Aβ1-42/phospho-tau levels in the cerebrospinal fluid (CSF) were used for confirmation. According to current guidelines for the diagnosis of mild cognitive impairment due to Alzheimer’s disease^28^, the patients all underwent medical, neurological, neuropsychological and neuroradiological examinations. They had subjective and caregiver-rated memory complaints, confirmed by neuropsychological memory evaluations, as well as a Clinical Dementia Rating Scale (CDR) memory score of 0.5, isolated episodic memory deficits (< 1.5 SD of the normal mean for age and education), and normal performances in other areas of cognition and in global cognition (assessed with the MMSE scales)^18^.

### ^1^H-MRS acquisition and analysis

**Figure 1:**
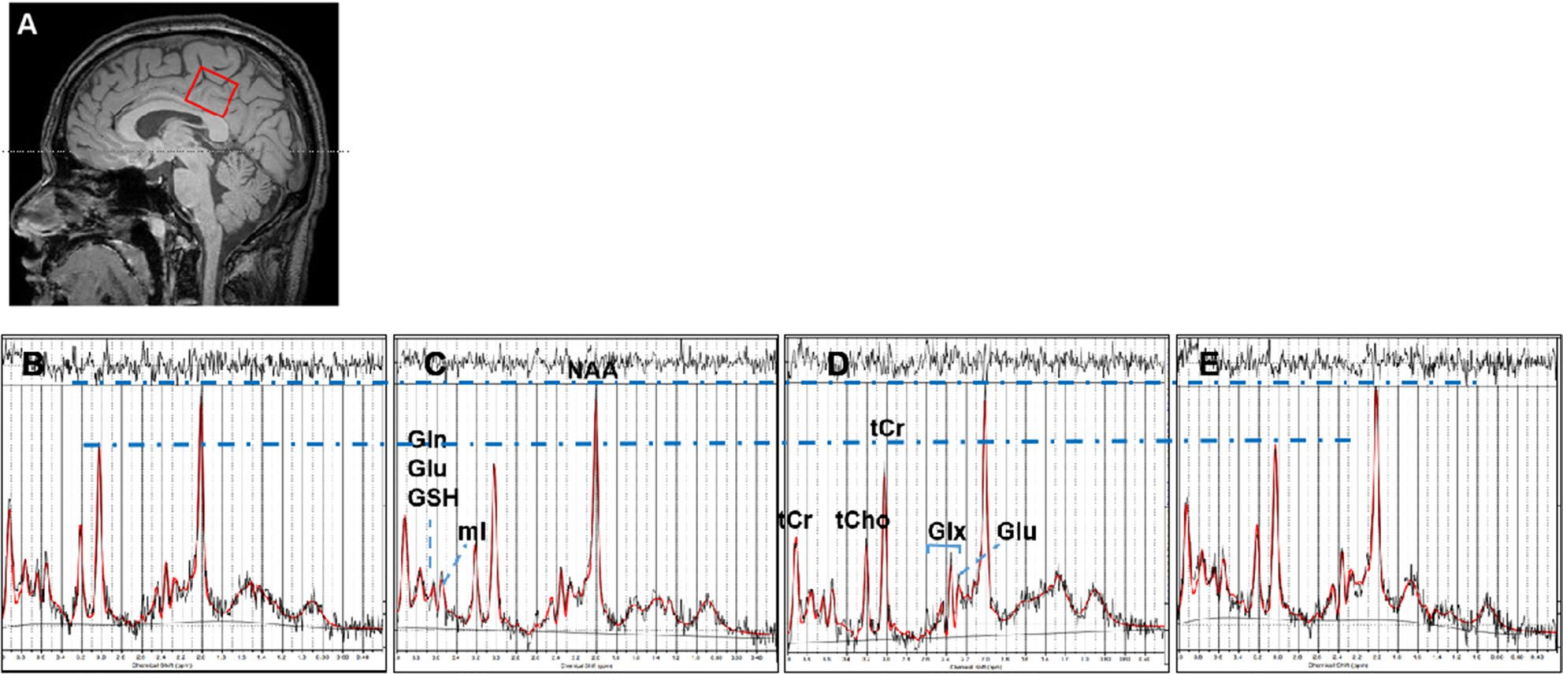
(**A**) T1 sagittal MRI showing the voxel of interest within the posterior cingulate gyrus. Representative spectra from study participants are shown. (**B**) Healthy control participant (HC). (**C**) PD patient with normal cognition (PDN). (**D**) PD patient with cognitive impairment (PDMCI). (**E**) Patient with cognitive impairment (PDMCI). *NAA* *N*-acetyl aspartate, *tCho* glycerophosphocholine + phosphocholine, *tCr* total creatine, *Ins (mI)* myoinositol, *GSH* Glutathione, *Glx* glutamate + glutamine.

^1^H-MRS utilized a 3.0 Tesla system (Philips Medical Systems, Achieva, Netherlands), with an 8-channel, head-neck-type coil. To avoid artifacts from head movements, the subjects were required to remain still and calm. Data were collected from a 2 × 2 × 2 cm^3^ voxel of interest (VOI) in a portion of the PCC. Data acquisition applied PRESS sequences with 2048 samples, a spectral band width of 2000 Hz, and 128 acquisitions, using a TR/TE of 2000/35 ms, and for total creatine (tCr), *N*-acetyl aspartate (NAA), choline (tCho), myoinositol (Ins), glutathione (GSH), glutamate (Glu) and glutamate + glutamine (Glx) (Fig. 1).

Metabolite concentrations were assessed with the LcModel/LCMgui package (Version 6.3, Steven Provencher, Oakville, ON, Canada). The concentrations of neurometabolites were estimated by fitting the obtained spectrum to a linear combination of the 'basic spectrum’ of each neurometabolite, using the LcModel software with a 3T PRESS acquisition at a TE of 35 ms. Using the latter software, Gaussian-fitted peak areas were determined relative to a baseline obtained from a moving average of noise regions for each spectrum. Signals within the chemical shift range of 0.0–4.0 ppm were obtained. Only the fitting results of Cramér–Rao lower bound < 15% were considered. For further analysis, the concentrations of neurometabolites expressed as the ratios of NAA and Cho to Cr, respectively, in the PCC were utilized.

### Statistical analysis

Continuous data are mean ± standard deviation. Statistical Package for the Social Sciences (SPSS; version 13, IBM). Continuous and dichotomous demographic variables were compared by the independent samples t-test and the chi-square test, respectively. Analysis of covariance (ANCOVA) was performed to compare metabolite profiles among groups. Homogeneity of variance was tested by the Bonferroni test: if p ≥ 0.05, Bonferroni correction was used; otherwise, Dunnett T3 correction was adopted. Because of a significant reduction of tCr in the PDMCI group, elevated Ins and/or reduced NAA/Ins ratio may be risk markers of preclinical AD. We also applied receiver operating characteristic (ROC) curve analysis to assess the ability of tCr to distinguish PDMCI cases from PDN patients, and to assess the abilities of Ins and NAA/Ins ratio for differentiating between MCI cases and individuals with normal cognitive function. *p* < 0.05 was considered statistically significant.

### Ethical approval and consent to participate

All patients provided written informed consent. The study was approved by the Ethics Committee of the Affiliated Hospital of GuiZhou Medical University, and followed the Declaration of Helsinki.

## Results

**Figure 2:**
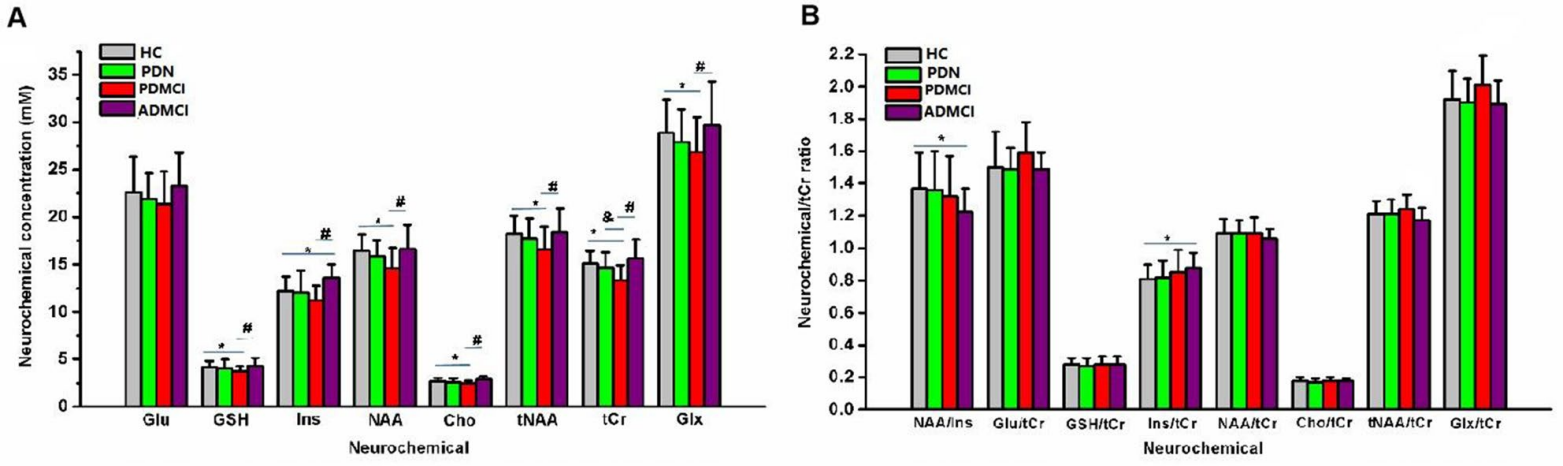
Metabolite concentrations (**A**) and metabolites/tCr ratios (**B**) of the PCC among four groups. (**A**) Compared with HCs, significantly decreased GSH, NAA, tNAA, Cho, tCr and Glx were detected in the PDMCI group (*p < 0.05). Significantly elevated levels of Ins and reduced NAA/Ins ratio were found in the ADMCI group. No changes were found in the PDN group. Compared with the PDN group, significantly reduced tCr was detected in the PDMCI group (^&^*p* < 0.05). Compared with the ADMCI group, significantly reduced GSH, Ins, NAA, tNAA, tCr and Glx and elevated Ins levels were detected in the PDMCI group (^#^*p* < 0.05). (**B**) According to metabolite concentrations (**A**), due to the varying concentration of tCr in PD progression, tCr is not suitable as an internal control metabolite. Therefore, less useful information was found among the HC, PDN and PDMCI groups from ratio analyses. However, in the ADMCI group, tCr remained stable and could be used as an internal control metabolite in ^1^H-MRS. Compared with HCs, significantly elevated Ins/tCr levels and reduced NAA/Ins were detected in ADMCI patients (*p < 0.05).

Compared with HCs, patients with PDMCI showed significantly reduced concentrations of NAA and tCr, while ADMCI cases had significantly increased amounts of Ins and reduced NAA/Ins ratio. NAA, Ins and tCr amounts were reduced in PDMCI compared with ADMCI. No significant metabolic alteration was found in PDN cases (Fig. 2A,B). Other metabolites, including GSH, Cho, Glu and Glx, were also detected: compared with healthy controls, PDMCI patients had decreased GSH, Cho and Glx levels in the PCC. No metabolic alterations were found in either PDN subjects or the ADMCI group. According to metabolite concentrations (Fig. 2A), due to varying concentrations of tCr in PD progression, tCr is not a suitable internal control metabolite. From ratio analysis, less useful findings were found among the HC, PDN and PDMCI groups. However, in the ADMCI group, tCr remained stable and can be used as an internal control metabolite in ^1^H-MRS. Compared with HCs, ADMCI patients had significantly elevated Ins/tCr and reduced NAA/Ins (Fig. 2B).

**Figure 3:**
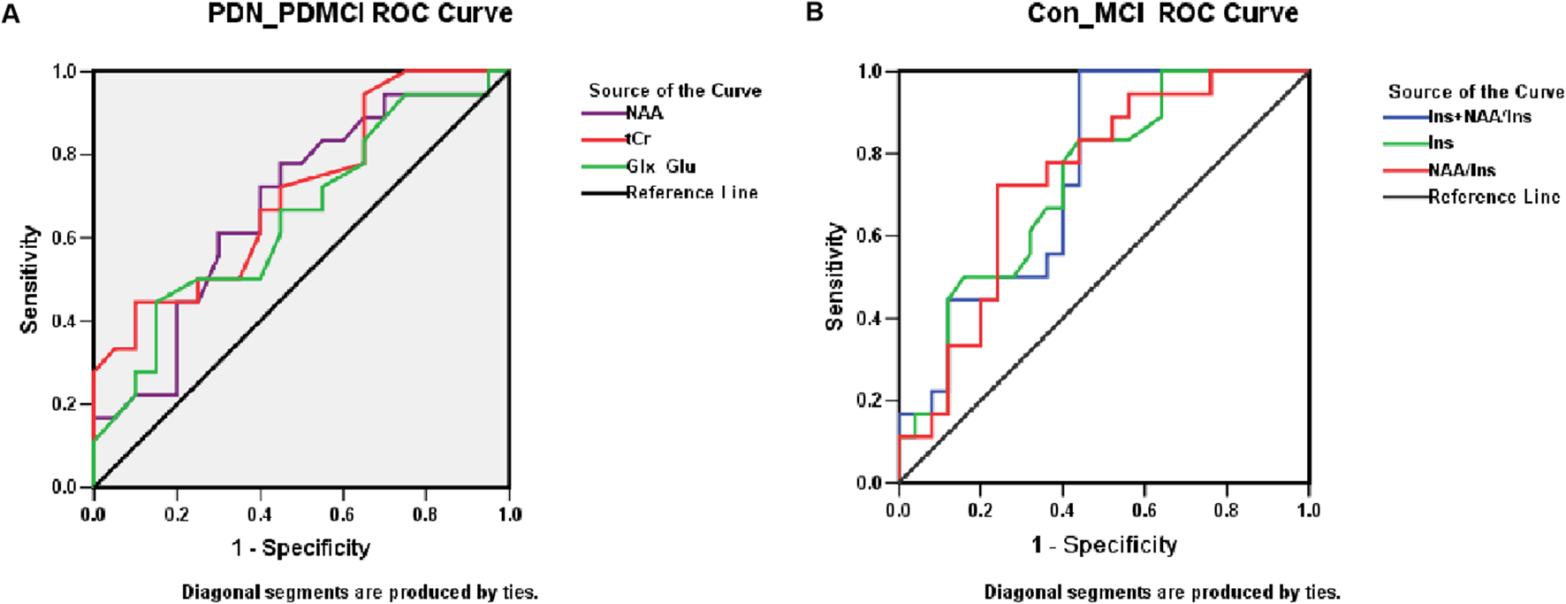
ROC curve analysis of metabolites and metabolic ratios. (**A**) PDN versus PDMCI. (**B**) Normal cognition versus ADMCI.

Additionally, in ROC curve analysis (Fig. 3), tCr could differentiate between PDMCI and PDN with an AUC of 0.71, a sensitivity of 44.4%, and a specificity of 90.0%, with a cutoff of < 15.45 mM/ml (Table 2). Furthermore, NAA/Ins ratio could differentiate between MCI cases and normal cognitive controls with an AUC of 0.74, a sensitivity of 72.2%, and a specificity of 76.0%, with a cutoff value of < 0.84 (Table 3).

**Table 2:** ROC curve analysis of neurochemical concentration (Ins) and neurochemical concentration ratio (NAA/Ins).

Neurochemical	Accuracy	Cutoff	Sensitivity	Specificity	p value
Ins	0.731	12.35	0.833	0.560	**0.010**
NAA/Ins	0.740	0.84	0.722	0.76	**0.008**
Ins + NAA/Ins	0.747	–	1.000	0.560	**0.006**

Bold values are statistically significant results.

**Table 3:** ROC curve analysis of neurochemical concentration and neurochemical concentration subtract between Glx and Glu.

Neurochemical	Accuracy	Cutoff	Sensitivity	Specificity	p value
NAA	0.679	14.80	0.778	0.550	0.059
tCr	0.707	15.45	0.444	0.900	**0.029**
Glx-Glu	0.650	6.250	0.444	0.850	0.114

Bold values are statistically significant results.

## Discussion

Mild cognitive impairment (MCI) precedes both Alzheimer’s disease (AD)- and Parkinson’s disease (PD)-associated dementia^2–4,13^. This is the first comparative study to examine metabolic patterns in the PCC (via single voxel ^1^H-MRS) between these two commonly neurodegenerative diseases. In the current study, the main findings were as follows. (1) Among the groups investigated, only the PCC in ADMCI cases showed significantly altered glial metabolism (Ins levels). (2) The levels of tCr were significantly reduced in the progression of cognitive impairment in PD, while in ADMCI, tCr remained unchanged. (3) Unlike ADMCI patients, individuals with PDMCI showed significantly reduced NAA, tCr, GSH, Cho and Glx levels in the PCC, but no changes were found in the PDN group. These findings suggest that ^1^H-MRS of the PCC demonstrate distinct metabolic brain abnormalities in potential PDMCI and ADMCI patients. There was no metabolic alteration in the PDN group, perhaps ^1^H-MRS of the PCC may be helpful for predicting cognitive impairment in PD progression. These findings may also suggest that different treatment strategies should be adopted for cognitive impairment between these two neurodegenerative disorders.

1. Different metabolic patterns in the PCC between PDMCI and ADMCI patients.

In the ADMCI group, the metabolic pattern of the PCC is well documented^12^, consisting of elevated Ins concentration or Ins/Cr ratio^13,14^. It has been suggested that Ins/Cr elevation in ADMCI possibly represents gliosis or indicates inflammation^1,29^. Many ^1^H-MRS studies in AD have detected reduced NAA and increased Ins, with less consistent findings for other metabolites^30^, and reduced NAA/Ins ratio is a potential biomarker for predicting progression to AD as proposed by Mitolo et al.^13^. We also found elevated Ins amounts and reduced NAA/Ins ratio in PCC in ADMCI cases. However, there was a significant reduction in NAA with no changes of Ins in PDMCI, consistent with the notion that NAA is a marker of functional integrity of neurons and emphasizes that PCC pathology is likely involved in PDMCI progression^26^. Specifically, NAA reduction in PDMCI is associated with neuronal loss, axonal injury and compromised neuronal energy metabolism, which was confirmed by other neuroimaging methods documenting gray matter atrophy^20^, abnormal white matter integrity^31^ and lower CBF perfusion^32^ in the PCC of PD patients with cognitive impairment.

In addition to NAA reduction, tCr was also significantly decreased in the PDMCI group, and these changes were found in neither ADMCI nor PDN. Cr is considered a biomarker of energy metabolism, which is typically used as an internal control metabolite in ^1^H-MRS^10,30^. Early in 2002, Neill et al.^33^ found a 24% loss of Cr in the substantia nigra (SN), and suggested loss of neurons and/or glia in the SN in PD. Therefore, putative changes in Cr in PD argue against Cr use as an internal reference in the quantitation of ^1^H-MRS-derived metabolite peaks; additionally, Cr may differ between gray and white matters, Cr varies regionally across the brain, and Cr changes with normal aging^34,35^. Our results also suggested that Cr amounts varied during PD progression and is not suitable as an internal control metabolite. So, there were less consistent findings for assessing metabolites by ^1^H-MRS in tracking cognitive decline in PD, may be due to using Cr as an internal control metabolite. Otherwise, in ADMCI, Cr levels remained stable during AD progression. Unlike AD, single voxel ^1^H-MRS of the PCC failed to show a significant association with cognitive status at baseline or over time. In the current study, we also adopted tCr ratio for investigating metabolic changes in the PCC among groups, but less useful information was found (Fig. 2B).

Due to improved signal-to-noise ratio and the utilization of the LCmodel software, besides NAA, Cr and Ins, other metabolites such as GSH, Cho, Glu and Glx can also be detected in this study. Compared with HCs, PDMCI patients had reduced GSH, Cho and Glx in the PCC, and these changes were not found in ADMCI or PDN. GSH is an endogenous antioxidant that affects many cellular functions^36,37^. Iskusnykh^36^ revealed that impaired GSH function in the brain is linked to neuronal loss during the aging process or as a result of neurological diseases, including Huntington’s disease, Parkinson’s disease, stroke, and Alzheimer’s disease. Sian et al.^38^ also reported altered GSH levels in PD. Cho is a marker of both membrane catabolism and anabolism. Cao et al.^39^ demonstrated that Cho/Cr in the substantia nigra is associated with PD severity, without mentioning cognitive impairment. Nie et al.^26^ reported that elevated Cho/Cr ratio in the PCC is associated with PDMCI, and suggested that Cho/Cr ratio may be used as a marker of PDMCI. The role played by Glu in idiopathic PD remains somewhat elusive^40,41^, and previous studies applying ^1^H-MRS in PD have not observed metabolic abnormalities in Glu, which may be because the studies were conducted with 1.5 T MRI scanners or adopted Glu/Cr ratio for reflecting Glu alteration (while Cr varies during PD progression as mentioned above). So, ^1^H-MRS at 3 T or higher magnetic field strengths should be applied in future investigation to track the course of metabolic brain changes in association with disease progression in PD cases. In this study, we also found Glx reduction in the PCC of PDMCI patients, corroborating Griffith et al., who reported Glu level reduction in the cerebral cortex of PD patients.

PDMCI and PDN patients showed distinct PCC metabolic ^1^H-MRS profiles. Compared with controls, tCr, NAA, Ins, GSH and tCho levels were significantly decreased in the PCC in the PDMCI group, while no significantly changes were found in the PDN group, with a slightly downward trend observed. Previous studies have reported no metabolite differences between PD patients and control subjects in either metabolite ratios or absolute concentrations of NAA, Cho, and Cr in various brain regions^9,27,42–44^. Significant alterations in neurochemical levels may provide evidence to elucidate the pathophysiological mechanisms underlying of PD^11^. This suggests there are no serious neuronal degenerations in the early onset of PD in cases with normal cognition. These results corroborated other studies reporting changes of NAA and Cho levels in the early cognitive impairment phase of PD^45^, and lower NAA/Cr ratio in the occipital lobe in PD patients with mild cognitive impairment^26^. The aforementioned metabolic abnormalities in the PCC in PDMCI may be due to lower regional cerebral blood flow in this region compared with PD with normal cognition, as revealed by Hosokai et al.^21^. Therefore, this study suggests that a comparison of PCC ^1^H-MRS profiles across mild cognitive impairment provides useful information for tracking cognitive decline in PD patients and can provide a window for potential therapeutic intervention in PDMCI.

Besides, ROC curve analysis revealed the absolute tCr concentration could differentiate between PDMCI and PDN with an AUC of 0.71, a sensitivity of 44.4%, and a specificity of 90.0%, with a cutoff of < 15.45 mM. Meanwhile, NAA/Ins ratio could differentiate subjects with MCI from controls with normal cognitive function with an AUC of 0.74, a sensitivity of 72.2%, and a specificity of 76.0%, with a cutoff of < 0.84. These findings were in line with Waragai et al.^14^, who found an AUC of 0.78, a sensitivity of 67.9%, and a specificity of 67.9% between the remaining normal versus progressor MCI groups. Future studies should take into consideration other neuroimaging indicators (hippocampus volume, diffusion tensor parameters and cortical thickness) to improve the sensitivity and specificity in discriminating between PDMCI and PDN, as well as ADMCI and healthy controls with normal cognitive function. This work proposes a promising biomarker that may possibly predict early PD cognitive impairment.

(2) Metabolic patterns in PDMCI and ADMCI

In ADMCI, the metabolic pattern of the PCC is well documented^12–14^, consisting of increased Ins and reduced NAA/Ins ratio; increased Ins in ADMCI possibly represents gliosis or neuroinflammation, although the underlying mechanism remains undefined. A prior comparison of AD with other dementia forms found Ins elevation only in patients with AD, while reduced NAA was not specific to AD^46^. This finding was consistent with prior reports demonstrating that Ins is not increased in PDMCI (and PDN), possibly suggesting that the degree of gliosis or inflammation is different in PDMCI (and PDN) compared with ADMCI.

Unlike ADMCI cases, PDMCI patients showed significantly reduced NAA and tCr. These findings were consistent with the notion of NAA as a marker of functional integrity in neurons, and Cr is usually considered a marker of energy metabolism, also emphasizing that posterior cortical pathology is likely implicated in PDMCI. According to Braak’s hypothesis^47^, PCC regions showed atrophy during PD progression, possibly indicating neuronal dysfunction and energy metabolism abnormalities in the progression of cognitive decline in PD. In ADMCI patients, different degrees of neuronal dysfunction and energy metabolism abnormalities are present.

Few studies have reported brain metabolism differs between Alzheimer disease and Parkinson disease dementia (PDD)^46^. Additionally, brain oscillatory patterns differed between ADMCI and PDMCI patients in a EEG study^48^. However, there is no comparative study examining metabolism between these two MCI forms. Therefore, in this study, comparing the ^1^H-MRS profiles of the PCC to examine cognitive impairment between PDMCI and ADMCI may provide useful information for better defining the disease process and elucidating possible treatment.

However, the current study had several limitations. First, single voxel ^1^H-MRS of the PCC was applied in this study, and other key brain regions related to cognitive impairment in PD were not taken into consideration; future studies should adopt multi-voxel ^1^H-MRS methods to detect additional neurobiomarkers. Secondly, the sample size was relatively small, and PD patients with dementia were not included; future studies should track PD’s cognitive status from normal cognitive to dementia. Thirdly, this was a single-center study, with heterogeneity in patient characteristics, including disease duration, disease severity, cognitive status and other non-motor clinical features, not clearly classified; therefore, further multi-center studies are required to investigate the different stages of PD progression. Fourthly, morphological changes of brain structure in PD have close relationships with cognitive deterioration, and thus may determine the progression of the disease. In future longitudinal studies, metabolites with other morphological parameters should be combined to track cognitive decline during PD progression.

## Conclusion

The ^1^H-MRS technology and the LcModel software were used to compare the metabolite profiles of the PCC between PDMCI and ADMCI patients. Unlike ADMCI patients, PDMCI subjects showed significantly reduced NAA, tCr and Ins, while ADMCI showed elevated Ins levels and reduced NAA/Ins ratio, but no changes were found in the PDN group. Therefore, metabolic abnormalities in the PDMCI group may be due to neuronal dysfunction in PD progression and results in cognitive decline in PD cases. Meanwhile, more serious gliosis or inflammation may exist in ADMCI compared with PDMCI. Increased Ins and reduced NAA/Ins ratio may be risk markers of preclinical Alzheimer's Disease; as in ADMCI patients, tCr levels could differentiate PDMCI from PDN, possibly suggesting that ^1^H-MRS of the PCC may be helpful in predicting cognitive impairment in PD progression.

## Data Availability

The datasets generated in the current study are available from the corresponding author on reasonable request.
